# Dynamic Digital Channelizer Based on Spectrum Sensing

**DOI:** 10.1371/journal.pone.0136349

**Published:** 2015-08-26

**Authors:** Junpeng Hu, Zhen Zuo, Zhiping Huang, Zhi Dong

**Affiliations:** School of Mechatronics Engineering and Automation, National University of Defense Technology, Changsha, Hunan Province, China; Glasgow University, UNITED KINGDOM

## Abstract

The ability to efficiently channelize a received signal with dynamic sub-channel bandwidths is a key requirement of software defined radio (SDR) systems. The digital channelizer, which is used to split the received signal into a number of sub-channels, plays an important role in SDR systems. In this paper, a design of dynamic digital channelizer is presented. The proposed method is novel in that it employs a cosine modulated filter bank (CMFB) to divide the received signal into multiple frequency sub-bands and a spectrum sensing technique, which is mostly used in cognitive radio, is introduced to detect the presence of signal of each sub-band. The method of spectrum sensing is carried out based on the eigenvalues of covariance matrix of received signal. The ratio of maximum-minimum eigenvalue of each sub-band is vulnerable to noise fluctuation. This paper suggests an optimized method to calculate the ratio of maximum-minimum eigenvalue. The simulation results imply that the design of digital channelizer can effectively separate the received signal with dynamically changeable sub-channel signals.

## Introduction

The concept of software defined radio (SDR) means that the same hardware architecture can be programmed or reconfigured to cope with any radio standard [1]. The digital channelizer is used to deal with the SDR signal composed of a large number of frequency division multiplexed (FDM) channels. It is employed to extract individual sub-channels from a digitized wideband input signal [2]. By extracting multiple narrowband sub-channels from the wideband input signal, it can efficiently reduce the sampling rate and bring convenience for the further base-band processing. The received signal of SDR system usually appears with several sub-channel signals with dynamically changeable bandwidths and band locations [3]. It leads to the requirement of dynamic digital channelizer.

A conventional way to realize channelization is feeding the received signal to a filter bank (FB). Many research achievements have been presented in recent years. The per-channel (PC) structure extracts each distinct sub-channel signal using a separate digital filter. It needs the awareness of the bandwidth of each sub-channel. Discrete Fourier transform filter bank (DFTFB) is an efficient kind of FB which is being widely used in SDR communication. But the DFTFB can not deal with nonuniform sub-channels and can not extract channels with distinct bandwidths simultaneously [4]. A design of channelizer for multi-standard SDR receivers is introduced in [1], which referred to FRM FB and coefficient decimation based FB. But it is not applicable to realize dynamic channelizer. A wideband channelizer for SDR systems using modulated FB has been proposed in [5]. It consists of an analysis section and a synthesis section. Given the minimum width of guard-band, the method mentioned in [5] can theoretically realize dynamic channelization. A nonuniform FB obtained by merging the corresponding filters of a uniform cosine modulated filter bank (CMFB) is proposed in [6]. However, all the methods mentioned above do not give a way to detect the existence of signal.

Spectrum sensing is a key technique in cognitive radio. Its task is to obtain awareness about the spectrum usage in certain frequency band [7]. Many spectrum sensing methods have been proposed, such as energy detection [8, 9], match filtering [10], cyclostationary detection [11], and eigenvalue-based detectors [12]. The match filtering method needs the prior knowledge of received signal and synchronization, which is impractical in blind signal process. The cyclostationary detection method requires knowing the cyclic frequency and leads to a high complexity. Energy detection, though does not need any information about the received signal, requires knowledge of noise power and it is influenced easily by noise uncertainty.

A spectrum sensing method based on the difference between eigenvalues of signal and noise has been proposed in recent years [12–15]. It takes the maximum and minimum eigenvalue of the covariance matrix of received signal as the estimation of useful signal and noise power, respectively. It can overcome the shortcoming of energy detection and does not need any information of the signal and noise. It is a totally blind detection method [15].

This paper proposes a simple structure of digital channelizer which consists of analysis and synthesis section based on spectrum sensing technique. The analysis section divides the received signal into many frequency sub-bands using CMFB. The synthesis section reconstructs the final sub-channel signal from the sub-bands output, according to the existence of signal of each sub-band. The spectrum sensing method, which is employed to determine in which sub-band the signal truly resides, is designed based on the eigenvalue of the covariance matrix of received signal in this paper. To overcome the effect of noise uncertainty and fluctuation, the selection of the maximum and minimum eigenvalue is optimized. Some experiments are given to demonstrate the effectiveness of this proposed method.

The rest of this paper is outlined as follow: the proposed structure of digital channelizer is described in section 2. A review of modulated FB is presented in section 3. Section 4 mainly discusses the eigenvalue-based spectrum sensing method. Some experiment results are shown in section 5, followed with the conclusions in section 6.

## Proposed Structure of Digital Channelizer

There have been tremendous academic researches on the structure of digital channelizer [1–5, 16–19]. One of the widely used structure consists of an analysis section and synthesis section. The analysis section separates the received signal into several sub-bands. The output of each sub-band is decimated to reduce the sample rate. Then the synthesis section combines the adjacent sub-bands which contain the same sub-channel signal. Many previous works have achieved a remarkable performance using the analysis-synthesis structure. But few of them detail the method to detect the existence of signal in each sub-band.

**Fig 1:**
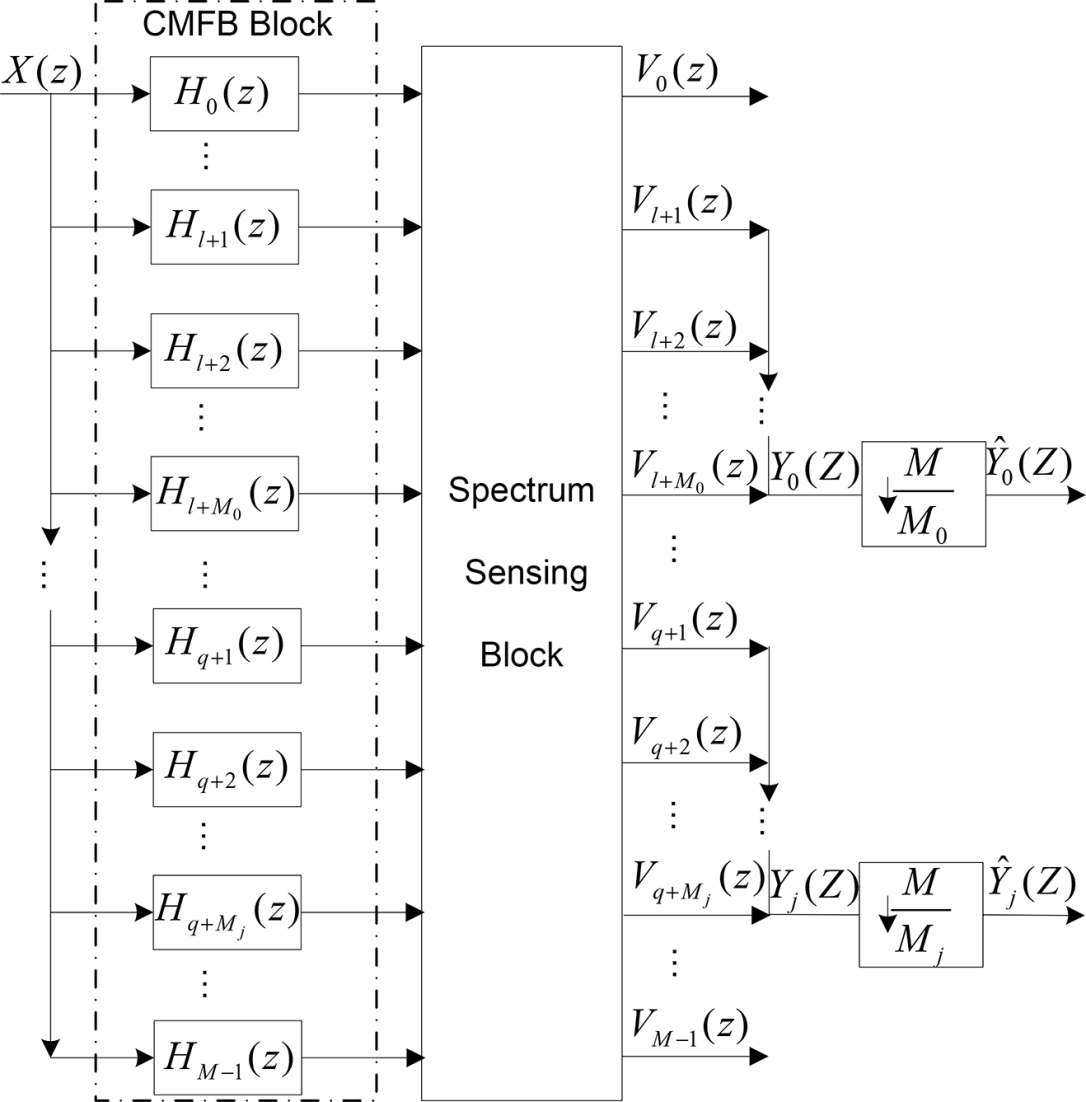
Proposed Structure of Digital Channelizer.

This paper proposes a simple structure of dynamic digital channelizer based on spectrum sensing as shown in Fig 1. The received signal *X*(*z*) is divided into *M* sub-bands by a CMFB. Since the bandwidth of each sub-band signal is 1/*M* of the received signal, each output of the CMFB is oversampled by the factor *M*.

$$V_{i}(z)=X(z)H_{i}(z),i=0,1,\cdots ,M-1$$ (1)

Instead of decimating immediately, the output data of each sub-band is directly fed to a spectrum sensing block to estimate the existence of signal. It can be found in the following section that the oversampled data is suitable rightly for the spectrum sensing method proposed in this paper. And then the adjacent sub-bands which contain signal are combined together, while the others will be ignored. The combined signal will be decimated, and the decimation factor is depending on the total number of combined sub-bands. In the real application, the structure can be further optimized by using a multistage decimation filter structure [20], but it is not the emphasis of this paper.

For the *j*th sub-channel signal, let $Q_{j}^{l}$ and $Q_{j}^{u}$ denote the lower and upper number of the included sub-bands. That is, the *j*th sub-channel signal can be reconstructed from the $Q_{j}^{u}-Q_{j}^{l}+1$ sub-bands data.

$$Y_{j}(z)=\sum_{p=Q_{j}^{l}}^{Q_{j}^{u}}V_{p}(z)=\sum_{p=Q_{j}^{l}}^{Q_{j}^{u}}X(z)H_{p}(z)$$ (2)

It can be concluded that the bandwidth of the *j*th sub-channel signal *Y*_*j*_(*z*) is 1/*M*_*j*_ of the received signal, where $M_{j}=M/\left(Q_{j}^{u}-Q_{j}^{l}+1\right)$. The needed sampling rate of the *j*th output can be reduced by decimation, which benefits the following base-band processing. Then a decimator is applied with decimation factor *M*_*j*_. The final expression of the sub-channel signal is:

$$Y_{j}\hat{(}z)=\frac{1}{M_{j}}X(z)\sum_{k=Q_{j}^{l}}^{Q_{j}^{u}}H_{k}(z)+\frac{1}{M_{j}}\sum_{l=1}^{M_{j}-1}\sum_{k=Q_{j}^{l}}^{Q_{j}^{u}}X(zW_{M_{j}}^{l})H_{k}(zW_{M_{j}}^{l})$$ (3)

The first term is desired signal, and the second is aliasing one. The decimation factor of each final output is different and dynamically changeable.

## Modulated Filterbank

A common approach to realize the uniform filter bank (UFB) is to modulate a prototype filter (PF). The PF is a linear phase finite-length impulse response (FIR) filter. Many methods have been proposed to design the PF with linear phase. Ambede et al [16] propose a coefficient decimation method to obtain required FIR filter. The required PF is formulated by using interpolated FIR technique and single variable bisection type optimization technique in [21]. An optimization algorithm to design linear phase FB is presented in [22], which refers to interpolation technique.

A vital issue to design the CMFB is deciding the value of sub-band number *M*. An optional approach is making assumption that the minimum guard-band of all sub-channels is given beforehand [5]. Let $\omega_{i}^{l}$ and $\omega_{i}^{u}$ denote the lower and upper frequency limits of the *i*th sub-channel signal *X*_*i*_(*z*). *G*_*i*_ is defined to be the width of the band between the *i*th and (*i* + 1)th channel and *G*_min_ is the minimum value among all *N* guard-bands.

$$G_{\text{min}}=\underset{i=0,1,\ldots ,N-1}{\text{min}}(\omega_{i+1}^{l}-\omega_{i}^{u})$$ (4)

Then the sub-band number *M* can be determined as follow.

$$M=2^{\left\lceil {\text{log}}_{2}\left(\frac{\pi }{G_{\text{min}}}\right)\right\rceil }$$ (5)

Unlike the application of transmultiplexers or sub-band coding system, the aim of the PF design is not perfect reconstruction, but pass-band flatness and linear phase. To keep the integrity of the signal, the pass-band flatness of the CMFB should be guaranteed. *h*(*n*) should be designed to meet the flatness condition, with a pre-specified tolerance *δ* and sub-band number *M*.

$$1-\delta \leq \left|H_{0}(e^{j\omega })\right|\;+\left|H_{0}(e^{j(\omega -\pi /M)})\right|\;\leq 1+\delta$$ (6)

**Fig 2:**
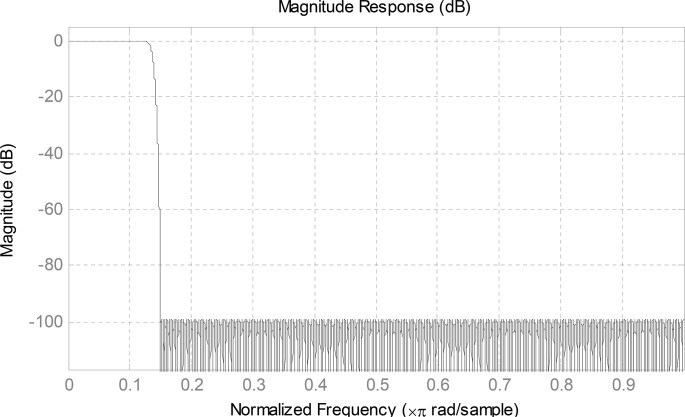
Magnitude Response of Designed Prototype Filter.

The magnitude response of the PF designed by Parks-McClellan algorithm with the objective function discussed above is shown in Fig 2. Then the CMFB can be implemented by modulating the PF with linear phase.

$$h_{k}(n)=2h_{0}(n)\;\text{cos}\left[\frac{\pi }{M}(k+0.5)\left(n-\frac{N-1}{2}\right)+{\left(-1\right)}^{k}\frac{\pi }{4}\right]$$ (7)

Where *N* is the order of *h*_0_(*n*), *n* = 0,1,…,2*mM* − 1, *k* = 0,1,…,*M* − 1. Then a CMFB with *M* branches is formed.

## Spectrum Sensing Method

The task of spectrum sensing is to detect the existence of signal in the related frequency band. There are many algorithms to realize the spectrum sensing, including the match filtering, cyclostationary detection and energy detection. In this section, a spectrum sensing method based on eigenvalues is given. It can overcome the shortcoming of energy detection and achieve blind detection. Assume that the received signal gets the following form.

x[n]=s[n]+w[n] (8)

where *s*[*n*] is the received signal to be detected, *w*[*n*] is the additive white Gaussian noise (AWGN) with mean zero and variance $\sigma_{n}^{2}$, and *n* is sample index. Note that *s*[*n*] = 0, when there is no signal contained in the receiver. Therefore, the spectrum sensing problem is equivalent to distinguishing between two hypotheses: *H*_0_, signal absent, and *H*_1_, signal present.

$$\{\begin{array}{l} H_{0}:x[n]=w[n]\\ H_{1}:x[n]=s[n]+w[n] \end{array}$$ (9)

It has been known from the former section that each sub-band data output is over-sampled at least by a factor *M*. Consequently, the oversampled method can be fittingly used to construct the covariance matrix. Assume that *x*_*i*_[*n*], *i* = 1,2,…,*M* denotes the output data of the *i*th sub-band, where *n* = 1,2,…,*L*. *L* is the length of the dataset and *L* = *NM*. A resampled data set can be defined as follow.

$$x_{i}^{j}[n]=x_{i}[nM+j-1],j=1,2,\ldots ,M$$ (10)

Where the subscript *i* denotes the number of sub-band signal, and superscript *j* denotes sample time of the resample signal. Then a data matrix **x**_*i*_ can be expressed as

$$x_{i}=[x_{i}^{1},x_{i}^{2},\cdots ,x_{i}^{M}]$$ (11)

The covariance matrices of each sub-band data are obtained.

$$R_{i}^{x}=E[x_{i}x_{i}{}^{T}]=x_{i}x_{i}{}^{T}/N,i=1,2,\ldots ,M$$ (12)

Assume that noise and signal are uncorrelated.

$$R_{i}^{x}=AR_{i}^{s}A^{T}+\sigma_{n}^{2}I$$ (13)

Where $R_{i}^{s}$ denotes the signal covariance matrix of the *i*th sub-band. *σ* ^2^ *I* represents the covariance matrix contains the noise. A spectrum sensing algorithm, based on the maximum-minimum eigenvalue, was proposed in [12]. $\lambda_{i}^{\text{max}}$ and $\lambda_{i}^{\text{min}}$ are the maximum and minimum eigenvalue of the matrix $R_{i}^{x}$. Then, the spectrum sensing decision can be made as follow: if $\lambda_{i}^{\text{max}}/\lambda_{i}^{\text{min}}>\alpha$, signal exists in the corresponding sub-band; otherwise, signal does not exist, where *α* > 1 is a threshold. The selection of the threshold *α*, is a key component of the spectrum sensing algorithms.

Let *P*_*d*_ be the probability of detection, and *P*_*f*_ be the probability of false alarm. the threshold *α* can be calculated by the follow [12, 14].

$$\alpha =\frac{{\left(\sqrt{N}+\sqrt{M}\right)}^{2}}{{\left(\sqrt{N}-\sqrt{M}\right)}^{2}}\cdot \left(1+\frac{{\left(\sqrt{N}+\sqrt{M}\right)}^{-2/3}}{{\left(NM\right)}^{1/6}}F_{1}^{-1}(1-P_{f})\right)$$ (14)

*F*_1_ is the cumulative distribution function of the Tracy-Widom distribution of order 1. The value of *F*_1_ can be looked up from tables [23].

In the application of channelizer, each sub-band signal is filtered by a bandpass filter. Moreover, the multipath propagation and fluctuation of the noise may cause remarkable effect on the eigenvaluses of each sub-band signal. For example, an infinitesimal $\lambda_{i}^{\text{min}}$ may appear in the *i*th sub-band. Then a small $\lambda_{i}^{\text{max}}$ will cause a ratio bigger than the threshold, though $\lambda_{i}^{\text{max}}$ is actually produced by noise and the sub-band holds no useful signal. The selection of $\lambda_{i}^{\text{min}}$ and $\lambda_{i}^{\text{max}}$ should take an overview consideration of the whole sub-band signals.

According to the discussion above, the eigenvalue-based spectrum sensing problem boils down to the two hypotheses.

$$\left\{ \begin{array}{ll} H_{0}:x[n]=w[n] & \;\lambda_{i}^{\text{max}}/\lambda_{i}^{\text{min}}\leq \alpha \\ H_{1}:x[n]=s[n]+w[n] & \;\lambda_{i}^{\text{max}}/\lambda_{i}^{\text{min}}>\alpha \end{array}\right.$$ (15)

$\lambda_{i}^{\text{max}}$ and $\lambda_{i}^{\text{min}}$ is corresponding to the signal component and noise of each sub-band, respectively. This paper presents a novel selection of $\lambda_{i}^{\text{max}}$ and $\lambda_{i}^{\text{min}}$ to optimize the detection performance. By dividing the received signal into *M* sub-bands, eigenvalues of each sub-band signal are obtained, $\lambda_{i}^{j},i=1,2,\cdots M,j=1,2,\cdots ,M$. Subscript *i* denotes the sequence number of sub-band, and superscript *j* is related to eigenvalue of the *i*th sub-band. ${\bar{\lambda }}_{i}$is defined as the average value of the *M* eigenvalues of the *i*th sub-band signal.

$${\bar{\lambda }}_{i}=\frac{1}{M}\sum_{j=1}^{M}\lambda_{i}^{j}$$ (16)

Then *M* maximum eigenvalues of each sub-band signal are collected. These *M* maximum eigenvalues can be regarded as the main component of the sub-bands output, either signal or noise. On the other hand, it is concluded from the design of analysis FB that at least one of the sub-bands contains noise only. Therefore, the minimum of the *M* average eigenvalues can be a reasonable estimate of the noise component within the received signal.

The ratio of maximum-minimum eigenvalue to determine the presence of signal for each sub-band can be optimized as follow.

$$\alpha_{i}=\frac{\underset{j=1,2,\cdots ,M}{\text{max}}\lambda_{i}^{j}}{\underset{i=1,2,\cdots ,M}{\text{min}}{\bar{\lambda }}_{i}},i=1,2,\ldots ,M$$ (17)

If *α*_*i*_ > *α*, there is signal resides in the *i*th sub-band. Otherwise, it contains noise only.

The steps to realize the dynamic digital channelizer can be summarized as follow.

1. Design a CMFB by modulating a linear phase PF. The sub-band number of the CMFB is decided by the minimum guard-band of the received signal, as expressed in Eq (5).
2. Divide the received signal into *M* frequency bands. The signal of each band is oversampled by a factor *M*.
3. Construct data covariance matrix of each sub-band signal, using the oversampled signal.
4. Detect the presence of useful signal in each band by eigenvalue-based spectrum sensing technique.
5. Combine the adjacent bands which contain signal. Decimate the signal according to the combined sub-band number.

## Simulation

**Fig 3:**
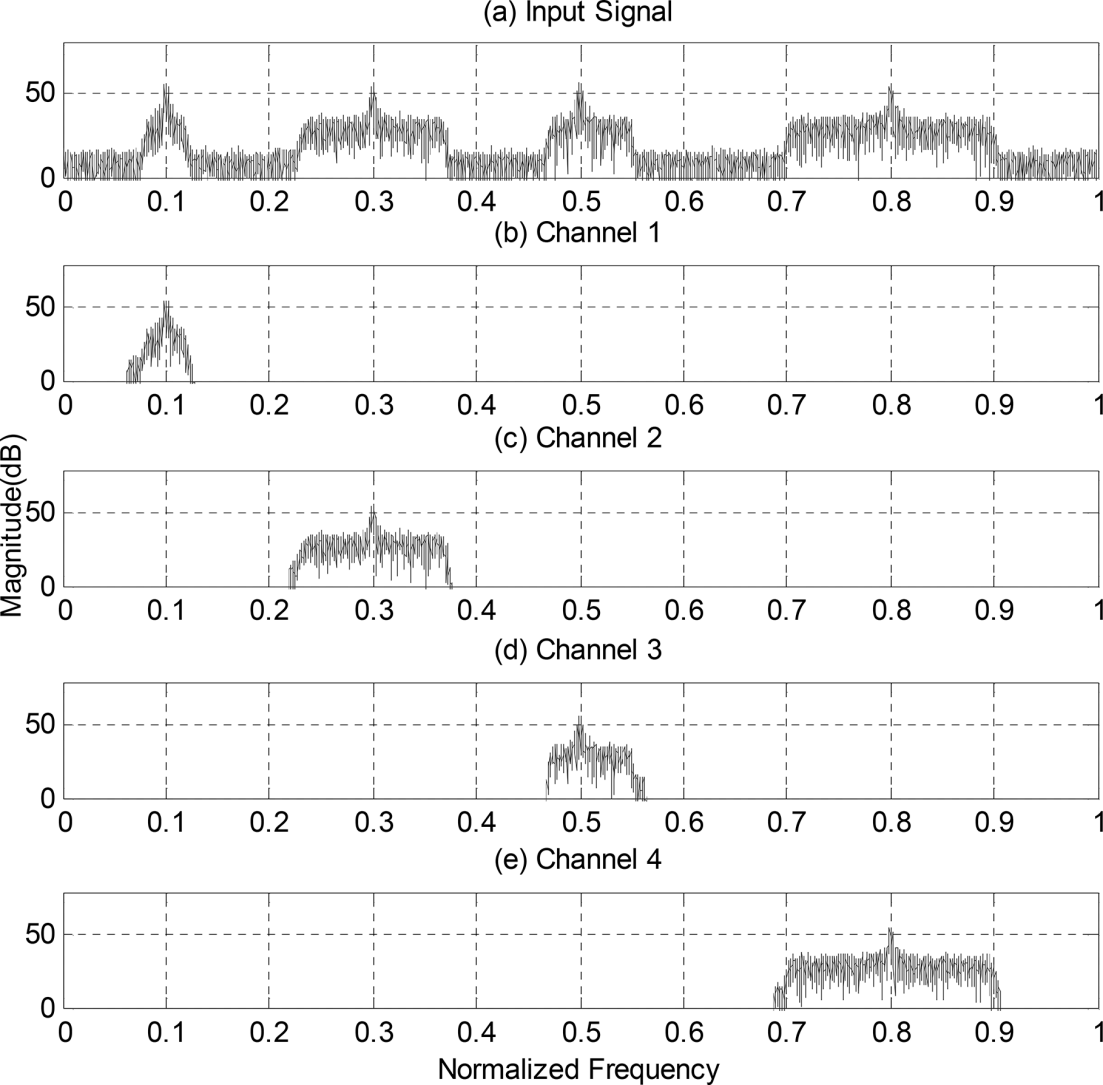
(a) Spectrum of Received Signal Containing 4 Sub-channels, (b)-(e) Channelized Signals Using Proposed Structure.

The functionality of the proposed structure of digital channelizer is verified in this section. A received signal with 4 sub-channels is shown in Fig 3(A). The received signal is divided into *M* = 16 sub-bands by CMFB, determined by the minimum guard-band. Then the detection of the existence of signal in each sub-band is implemented by eigenvalue-based spectrum sensing method.

The false alarm probability is set to *P*_*f*_ = 0.05. According to Eq (14), the detection threshold is *α* = 1.29. And the radio used to detect the existence of signal of each sub-band is depicted in Table 1.

**Table 1:** Ratio of maximum-minimum eigenvalue of each sub-band data of the received signal in Fig 3(A).

*Band number*	*1*	*2*	*3*	*4*	*5*	*6*	*7*	*8*
*ratio*	*1*.*07*	*747*.*73*	*1*.*00*	*14*.*12*	*817*.*95*	*123*.*00*	*1*.*00*	*386*.*00*
*Band number*	*9*	*10*	*11*	*12*	*13*	*14*	*15*	*16*
*ratio*	*370*.*87*	*1*.*03*	*1*.*05*	*83*.*27*	*848*.*71*	*136*.*62*	*35*.*78*	*1*.*03*

It can be inferred from Table 1 that the 2nd, 4th~6th, 8th~9th and 12th~15th sub-bands contain the consecutive sub-band signal, respectively. Combine the adjacent bands, the channelization result can be found in Fig 3(B)–3(E).

**Fig 4:**
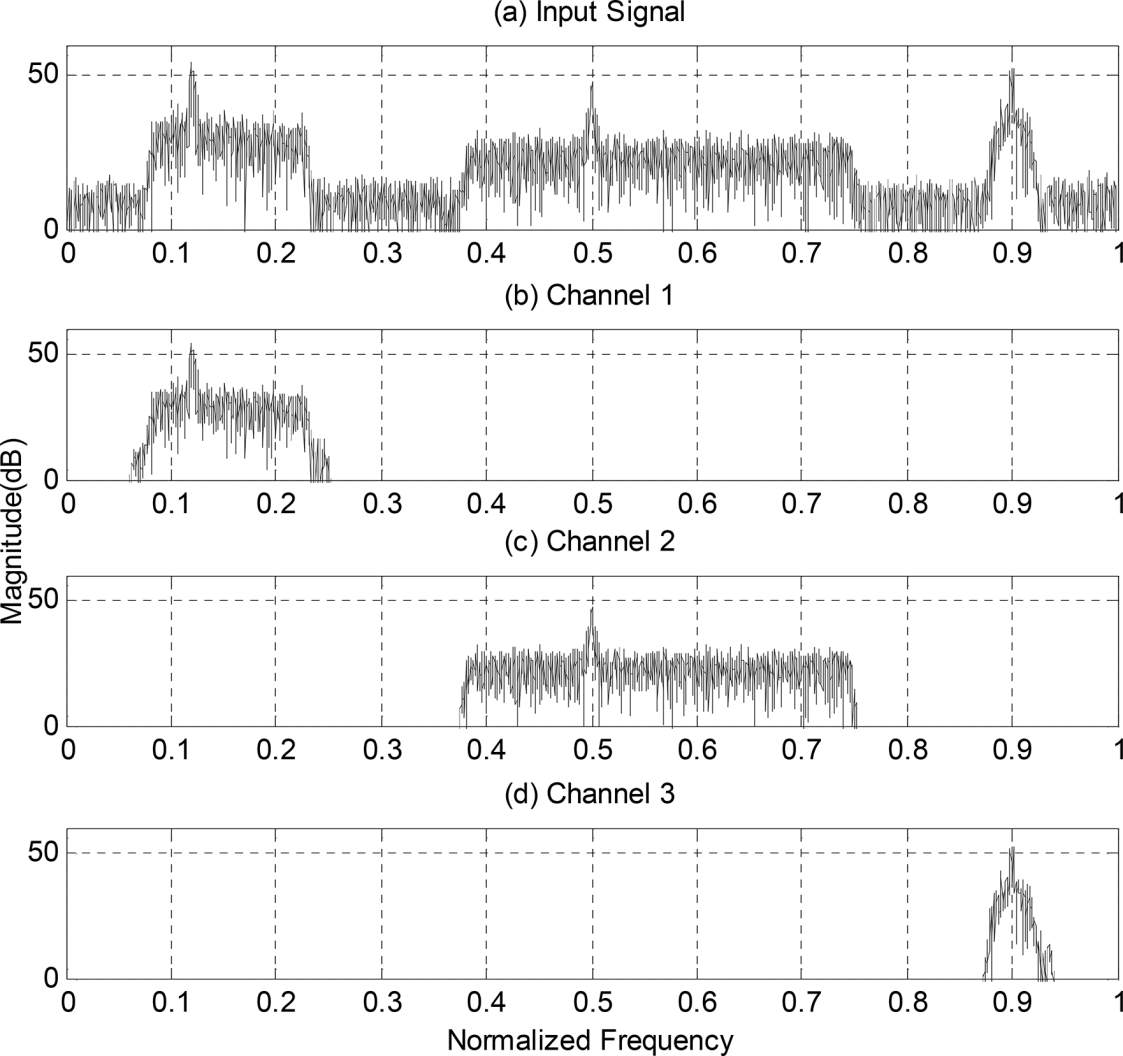
(a) Spectrum of Received Signal Containing 3 Sub-channels, (b)~(d) Channelized Signals Using Proposed Structure.

Another received signal with 3 sub-channels is depicted in Fig 4(A). The detection parameters are set the same as before. Then the 2nd~4th, 7th~12th and the 15th sub-bands are combined together to form the final sub-channel signal, concluded form Table 2. The results are shown in Fig 4(B)–4(D).

**Table 2:** Ratio of maximum-minimum eigenvalue of each sub-band data of the received signal in Fig 4(A).

*Band number*	*1*	*2*	*3*	*4*	*5*	*6*	*7*	*8*
*ratio*	*1*.*12*	*586*.*27*	*310*.*66*	*84*.*92*	*1*.*00*	*1*.*07*	*27*.*56*	*127*.*59*
*Band number*	*9*	*10*	*11*	*12*	*13*	*14*	*15*	*16*
*ratio*	*126*.*58*	*25*.*93*	*29*.*70*	*25*.*06*	*1*.*20*	*1*.*03*	*952*.*15*	*1*.*05*

The simulation results shown above indicate that the proposed structure of digital channelizer can split the received signal into multiple sub-channels effectively and the eigenvalue-based spectrum sensing technology can easily detect the existence of signal in each divided sub-band. The design of digital channelizer in this paper can fill the requirement of SDR system to dynamically channelize the received signal.

## Conclusions

A novel structure of dynamic digital channelizer, which consists of analysis and synthesis section as mentioned in the previous work, is proposed. The proposed structure introduces spectrum sensing technique to confirm the presence of signal in each band of analysis section. Unlike the previous work, the oversampled signals are fed to spectrum sensing section directly, instead of decimation. This process is fittingly convenient for the eigenvalue-based spectrum sensing. Due to requiring no prior knowledge of signal or noise, the eigenvalue-based method can realize blind spectrum detecting. Given the minimum guard-band of the received signal to determine the sub-band number of the modulated FB, the structure proposed in this paper can achieve dynamic digital channelizer for SDR systems.
